# Analysis of the prognostic value of uric acid on the efficacy of immunotherapy in patients with primary liver cancer

**DOI:** 10.1007/s12094-023-03314-2

**Published:** 2023-08-30

**Authors:** Hui Rao, Qi Wang, Xiaoli Zeng, Xuejiao Wen, Li Huang

**Affiliations:** 1 Department of Oncology, The First Affiliated Hospital of Gannan Medical University, No. 128, Jinling Road, Zhanggong District, Ganzhou, 341000 Jiangxi China; 2 The First Clinical Medical College, Gannan Medical University, Ganzhou, Jiangxi China; 3 Department of Hematology and Oncology, The First People’s Hospital of Nankang, Ganzhou, Jiangxi China; 4 Department of Health Statistics, School of Public Health and Health Management, Gannan Medical University, Ganzhou, Jiangxi China; 5 Jiangxi Clinical Medical Research Center for Cancer, Ganzhou, Jiangxi China

**Keywords:** Primary liver cancer, Immunotherapy, Uric acid, Efficacy, Biomarkers

## Abstract

**Purpose:**

Uric acid (UA) plays a dual role as an antioxidant and a prooxidant in patients with malignant tumors; however, the relationship between serum UA and malignancy is currently unclear. This study aims to investigate the prognostic value of serum uric acid level before immunotherapy on the efficacy of primary liver cancer (PLC) immunotherapy, which might provide a basis for optimizing the comprehensive treatment scheme.

**Methods:**

Patients with PLC who were admitted to the First Affiliated Hospital of Gannan Medical College from January 2019 to June 2022 and underwent immunotherapy were collected retrospectively. The difference between serum UA levels in patients with PLC, the correlation between serum UA levels, and the clinical characteristics of patients with PLC were analyzed using the chi-square test, and the survival was estimated using the Kaplan–Meier analysis. To further assess the prognostic significance of UA concentrations, univariate and multivariate Cox regression analyses were performed.

**Results:**

Ninety-nine patients were included in this study cohort. The median follow-up was 7 months (range: 1–29 months), and 76 (76.8%) of the 99 patients with PLC died as of December 31, 2022. Serum UA concentrations ranged from 105 to 670 μmol/l, with a median of 269 μmol/l. The results showed that the serum UA level of patients with PLC was higher than that of healthy subjects (*P* < 0.001). After subgroup analyses, only male patients with liver cancer had higher serum UA levels than healthy men (*P* = 0.001). The results of the Kaplan–Meier analysis showed that higher UA levels were associated with poor overall survival (OS) (*P* = 0.005). In univariate analysis, the OS rate of patients with elevated serum UA levels was significantly lower than the cut-off value (hazard ratio [HR]: 3.191, 95% confidence interval [CI]: 1.456–6.993,* P* = 0.004), with a median survival time of 151 and 312 days in the high and low serum UA groups, respectively. The results of multivariate analysis showed that the UA level was an independent prognostic factor for immunotherapy in patients with PLC (HR: 3.131, 95% CI: 1.766–5.553, *P* < 0.001).

**Conclusions:**

The serum UA level is a reliable biomarker for predicting the prognosis of patients undergoing immunotherapy for PLC, and might provide a basis for the individualized treatment of these patients. Dynamic monitoring of the serum UA level may compensate for the deficiency of the current liver cancer staging system.

## Introduction

Primary liver cancer (PLC), referred to as liver cancer, is one of the most common malignant tumors worldwide, with an incidence rate ranking fourth in China, after lung cancer, colorectal cancer, gastric cancer, and breast cancer, as well as the second highest mortality rate. PLC includes hepatocellular carcinoma (HCC), which comprises intrahepatic cholangiocarcinoma (ICC) and mixed types. Although surgical resection is the main treatment for PLC, most patients are diagnosed in the middle and late stages, at which point they have lost the opportunity for surgery and can only receive locoregional therapy or systemic treatment [1]. Systemic treatment is mainly targeted therapy, immunotherapy, and systemic chemotherapy, mainly oxaliplatin. With the continuous emergence of new immunotherapy methods, how to select the most suitable treatment method for PLC remains an urgent problem to be solved. Some studies have shown that tumor mutational burden (TMB), programmed death receptor ligand-1 (PD-L1), and microsatellite instability (MSI) are effective biomarkers for assessing the efficacy of immune checkpoint inhibitors (ICIs) [2]. In patients with advanced PLC, more research is needed to explore biomarkers to assist with the screening of people who can benefit from immunotherapy and avoid unnecessary cost, excessive progression, and possible serious toxicity in those who do not respond to treatment, achieving precision immunotherapy for malignant tumors.

Uric acid (UA) is produced by endogenous purine metabolism or dietary intake and is mainly excreted through the kidneys [3]. Elevated UA levels are associated with cardiovascular disease, metabolic syndrome, and kidney disease. Often, UA is not considered the cause but only an indicator of the disease. Recently, UA has received much attention as a potential biomarker. Higher UA levels can enhance inflammation, leading to gout and cardiovascular and kidney diseases, while UA also can chelate metal ions and scavenge free radicals [4]. The relationship between serum UA and malignancy is unclear, and increasingly high-quality studies are needed to evaluate its role in the risk of malignancy and the efficacy of antitumor therapy.

## Materials and methods

### Study population

**Fig. 1:**
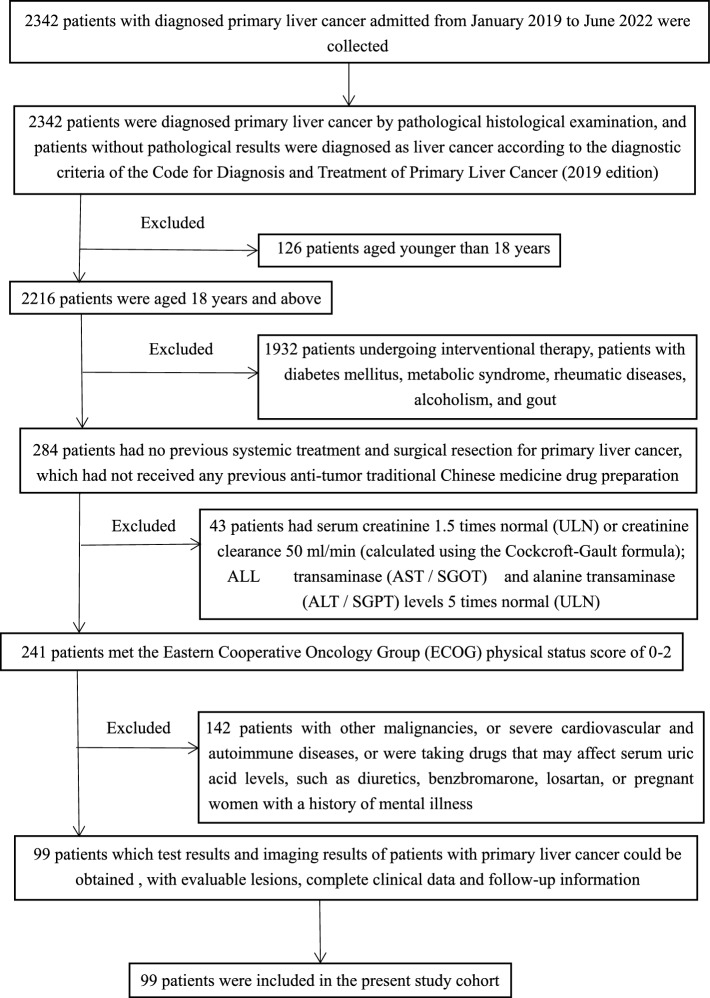
The flow diagram of the study participant selection

In this retrospective study, we collected 2342 patients who were diagnosed with PLC and were admitted to the First Affiliated Hospital of Gannan Medical College from January 2019 to June 2022. Enrolled patients were strictly screened according to the inclusion and exclusion criteria, and patients who failed screening were excluded from any analysis. Finally, 99 patients were enrolled in this study cohort, including 86 men and 13 women, aged 26–84 years, with a mean age of 57.79 years. The flow diagram of the study participant selection is shown in Fig. 1.

### Inclusion criteria

The inclusion criteria and selection process for the included patients with PLC were as follows:

I. Patients diagnosed with PLC by pathological histological examination. Patients with no pathological results, according to the diagnostic criteria for the Diagnosis and Treatment of Primary Liver Cancer (2019 edition) [1].

II. Age: ≥ 18 years.

III. No previous systemic treatment and surgical resection for PLC.

IV. Eastern Cooperative Oncology Group (ECOG) physical status score 0–2.

V. Available test and imaging results for patients with PLC.

VI. Patients with evaluable lesions and complete clinical and follow-up data.

### Exclusion criteria

The exclusion criteria and selection process for the included patients with PLC were as follows:

I. Patients undergoing interventional therapy with diabetes, metabolic syndrome, rheumatic diseases, alcoholism, gout, and other influential data sources.

II. Serum creatinine ≥ 1.5 times the upper limit of normal (ULN) or creatinine clearance ≤ 50 ml/min (calculated using the Cockcroft-Gault formula); aspartate transaminase (AST/SGOT) and alanine transaminase aminotransferase (ALT/SGPT) levels ≥ 5 times the ULN.

III. Patients with other malignancies.

IV. Patients with severe cardiovascular and cerebrovascular disease and autoimmune disease.

V. Patients taking medications that may affect serum UA levels, such as diuretics, benzbromarone, and losartan.

VI. Pregnant women and patients with a history of mental illness.

### Data collection

The following information was collected through the electronic medical record system of our hospital:

I. Demographic data: sex, age, height, weight, and ECOG score.

II. Previous history: hepatitis B infection, history of drinking alcohol, smoking history, history of concomitant diseases, and past medication history.

III. Tumor diagnosis: Barcelona Clinic Liver Cancer (BCLC) staging, Child–Pugh classification, China liver cancer classification (CNLC), liver cancer type, alpha-fetoprotein (AFP), liver tumor number, vascular invasion, lymph node enlargement, distant metastasis, portal hypertension, venous tumor embolism, liver cirrhosis, ascites, and splenomegaly.

IV. Treatment: immunotherapy drugs, the number of immunotherapy drugs, and targeted therapy.

### The selection criteria for patients with PLC who underwent immunotherapy

Within seven days of submitting the initial peripheral blood samples to our hospital, all of the enrolled patients got immunotherapy. After tight inclusion and exclusion criteria, only immunotherapy (containing PD-1 and PD-LL immune-checkpoint inhibitors with or without targeted therapy) was used as anti-tumor therapy on the research population. Of the 99 enrolled patients, 54 were treated with Camrelizumab, 23 were treated with Sintilimab, 15 with Tislelizumab and 7 with Toripalimab.

### Laboratory assays

Peripheral blood samples were collected during the first visit to our hospital, and all patients received immunotherapy within 7 days. Blood samples for biochemical measurements were obtained by standard venipuncture of the antecubital fossa vein (antecubital vein) on the morning after the subjects had fasted for 8 h. Serum UA was detected using a cobas e 801 automatic chemiluminescence immunoanalyzer with electrical chemiluminescence.

### Statistical analysis

Statistical analysis was performed using SPSS 23.0 software, and *P* < 0.05 was considered statistically different. Measurement data are summarized by the mean, standard deviation, median, minimum value, and maximum value; counting data are summarized by the frequency and percentage. The difference between serum UA levels in patients with PLC and the healthy population was analyzed by t-test. The optimal cut-off value and the area under the curve (AUC) were determined by drawing the receiver operating characteristic (ROC) curve. The correlation between serum UA levels and the clinical characteristics of patients with PLC was assessed. Survival was estimated using the Kaplan–Meier method using overall survival (OS) as the primary study endpoint, and the overall 95% confidence interval (CI) for the median time was calculated using the Brookmeyer Crowley method. Univariate analysis was performed using the Log-rank test to compare patient survival differences between the different groups. To exclude the influence of confounding factors, meaningful variables for univariate analysis were included in the multivariate Cox proportional model to analyze independent prognostic factors affecting OS in patients with PLC.

Processing of missing data: Enrolled patients who were still alive as of follow-up on December 31, 2022, were included in OS calculations as data deletions. The OS of data censoring was defined as the time from the start date of diagnosis to the censoring. The OS was measured in months or days.

## Results

### Patient characteristics

Enrolled patients were screened by strict inclusion and exclusion criteria, and a total of 99 patients diagnosed with PLC were included in this study cohort. The median follow-up period was 7 months (range: 1–29 months), and 76 (76.8%) of the 99 patients with PLC died as of December 31, 2022. The patient baseline demographics and lifestyle characteristics at diagnosis are summarized in Table 1. Among these patients, 86 were men and 13 were women, who ranged in age from 26 to 84 years, with a mean age of 57.79 years. Additionally, 92 had HCC, and seven had ICC. The patients were divided into four stages: seven cases in CNLC stage I, nine cases in CNLC stage II, 77 cases in CNLC stage III, and six cases in CNLC stage IV; BCLC staging: eight cases in A, seven cases in B, 79 cases in C, and five cases in D. The average number of immunotherapy treatments used was three. Patient serum UA concentrations ranged from 105 to 670 μmol/l, with a median value of 269 μmol/l.

**Table 1:** Baseline demographics and lifestyle characteristics of 99 patients with primary liver cancer

Characteristics	Group	n (99)	%
Sex	Male	86	86.87
	Female	13	13.13
Age (y)	≤ 50	26	26.26
	> 50	73	73.74
BMI (kg/m^2^)	> 23.9	16	16.16
	23.9–18.5	75	75.76
	< 18.5	8	8.08
Number of immunotherapy medications	1	46	46.46
	> 1	53	53.54
BCLC staging	A	8	8.08
	B	7	7.07
	C	79	79.80
	D	5	5.05
Child–Pugh classification	A	51	51.52
	B	40	40.40
	C	8	8.08
CNLC stage	I	7	7.07
	II	9	9.09
	III	77	77.78
	IV	6	6.06
PLC type	HCC	92	92.93
	ICC	7	7.07
Hepatitis B infection	Yes	76	76.77
	No	23	23.23
History of drinking	Yes	33	33.33
	No	66	66.67
History of smoking	Yes	36	36.36
	No	63	63.64
Immunotherapy drugs	Camrelizumab	54	54.55
	Other	45	45.45
Targeted therapy	Unite	74	74.75
	Asynapsis	25	25.25
Number of liver lesions	Single	42	42.42
	Pilosity	57	57.58
Vascular invasion	Yes	66	66.67
	No	33	33.33
Lymphadenectasis	Yes	49	49.49
	No	50	50.51
Distance transfer	Yes	46	46.46
	No	53	53.54
Portal hypertension	Yes	50	50.51
	No	49	49.49
Venous cancer emboli	Yes	65	65.66
	No	34	34.34
Hepatocirrhosis	Yes	75	75.76
	No	24	24.24
Ascites	Yes	52	52.53
	No	47	47.47
Splenomegaly	Yes	56	56.57
	No	43	43.43

### Differences in UA levels in patients with PLC and healthy populations

In 2020, the American College of Rheumatology set serum UA values for healthy men and women as 270 μmol/l (reference range: 120–420 μmol/l) and 240 μmol/l (reference range: 120–360 μmol/l), respectively [3]. Serum UA levels of patients with PLC were compared with the healthy population. The results showed that the serum UA level of patients with PLC was higher than that of the healthy population (95% CI: 29.52–73.25, *P* < 0.001). In the subgroup analysis by sex, only men with liver cancer had higher UA levels than healthy men (95% CI: 17.41–65.61, *P* = 0.001), while no correlation was found in women (*P* > 0.05).

### Correlation between UA and the clinical characteristics of patients with PLC

**Fig. 2:**
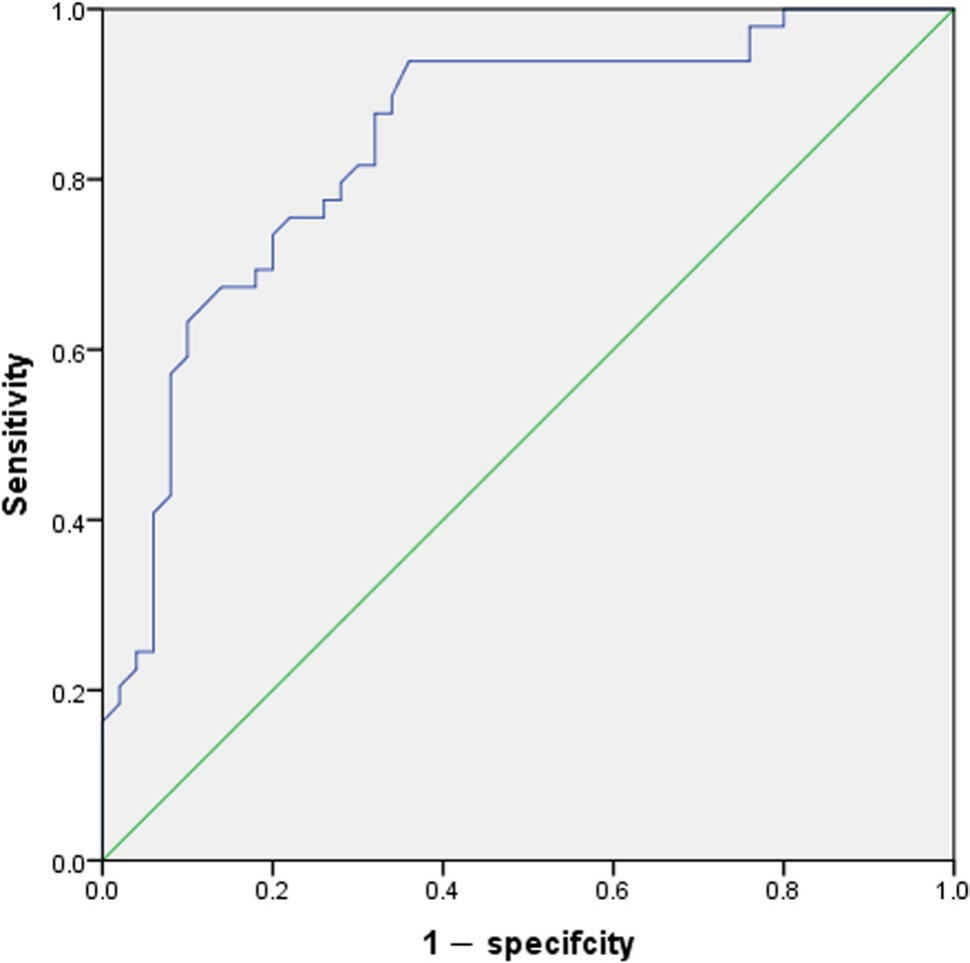
Receiver operating characteristic (ROC) curve analysis for uric acid in predicting the efficacy of immunotherapy in patients with primary liver cancer

The ROC curve of serum UA was used to determine the optimal cut-off concentration of serum UA and to predict the efficacy of immunotherapy based on the case data level and the efficacy of immunotherapy (the 6-month survival rate of patients with PLC was taken as the outcome) (Fig. 2). The value corresponding to the maximum Youden index is the best clinical cut-off value (threshold), and the corresponding AUC is calculated. The results showed that the optimal clinical cut-off value of serum UA was 259 μmol/l (specificity: 64.0%, sensitivity: 93.9%, and AUC: 0.843), and the Youden index was 0.579. Because the AUC was > 0.5, UA was considered to be valuable for predicting the efficacy of immunotherapy in patients with liver cancer. Further, the patients with PLC were divided into a low UA group (≤ 259 μmol/l) and a high UA group (> 259 μmol/l). The correlation between different levels of serum UA and the clinical characteristics of the patients is shown in Table 2. The results showed that UA levels were significantly correlated with body mass index (BMI; kg/m^2^, *P* = 0.012) and Child–Pugh classification (*P* = 0.006).

**Table 2:** Relationship between clinical characteristics and uric acid levels in patients with primary liver cancer before immunotherapy

Characteristics	Groups	Low uric acid group	High uric acid group	*P*-value
Gender	Male	30	56	0.767
	Female	5	8	
Age (y)	≤ 50	11	15	0.388
	> 50	24	49	
BMI (kg/m^2^)	> 23.9	8	8	0.012
	23.9–18.5	21	54	
	< 18.5	6	2	
Number of immunotherapy medications	1	19	27	0.249
	> 1	16	37	
BCLC staging	A	4	4	0.105
	B	3	4	
	C	24	55	
	D	4	1	
Child–Pugh classification	A	16	35	0.006
	B	12	28	
	C	7	1	
CNLC stage	I	3	4	0.385
	II	3	6	
	III	25	52	
	IV	4	2	
PLC type	HCC	33	59	0.697
	ICC	2	5	
Hepatitis B infection	Yes	26	50	0.665
	No	9	14	
History of drinking	Yes	10	23	0.457
	No	25	41	
History of smoking	Yes	14	22	0.578
	No	21	42	
Immunotherapy drugs	Camrelizumab	22	32	0.219
	Other	13	32	
Targeted therapy	Unite	28	46	0.374
	Asynapsis	7	18	
AFP	≥ 400	19	45	0.062
	< 400	16	19	
Number of liver lesions	Single	19	38	0.624
	Pilosity	16	26	
Vascular invasion	Yes	21	45	0.298
	No	14	19	
Lymphadenectasis	Yes	20	29	0.269
	No	15	35	
Distance transfer	Yes	12	34	0.072
	No	23	30	
Portal hypertension	Yes	15	35	0.256
	No	20	29	
Venous cancer emboli	Yes	21	44	0.381
	No	14	20	
Hepatocirrhosis	Yes	27	48	0.812
	No	8	16	
Ascites	Yes	19	33	0.795
	No	16	31	
Splenomegaly	Yes	17	39	0.235
	No	18	25	

### OS analysis of immunotherapy in patients with PLC

**Fig. 3:**
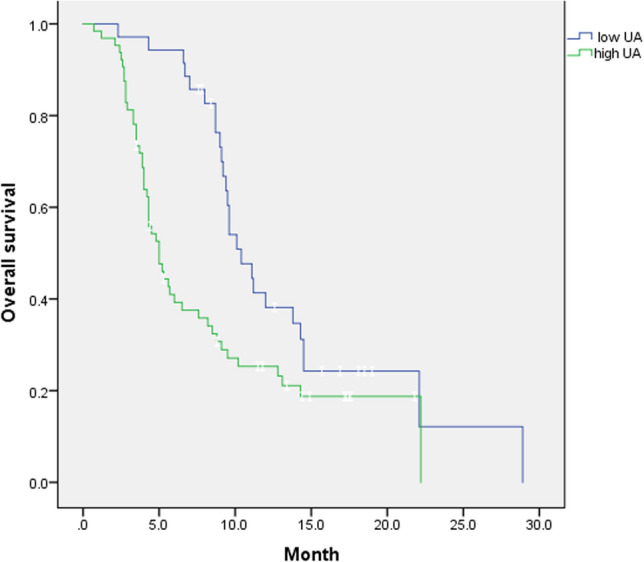
Kaplan–Meier analysis of overall survival according to uric acid levels, with low UA level (≤ 259 μmol/l) and high UA level (> 259 μmol/l)

During a mean follow-up period of 7 months (range: 1–29 months), 76 (76.8%) of the 99 patients with PLC died as of December 31, 2022. According to the ROC curve results, taking the UA cut-off of 259 μmol/l divided patients into the low UA group (259 μmol/l) and the high UA group (> 259 μmol/l), the Kaplan–Meier curve showed that high UA level was associated with worse OS before receiving immunotherapy (*P* = 0.005, log-rank test; Fig. 3). The median survival time was 151 days versus 312 days in the high serum UA group, and the accumulation of serum UA in vivo may lead to tumor progression and shortened patient survival.

### Regression analysis affecting the efficacy of immunotherapy in patients with PLC

To explore whether serum UA levels and various clinical variables were associated with the clinical outcomes of patients with PLC, we calculated OS using a univariate Cox ratio model (Table 3). In univariate analysis, the OS was significantly lower in patients with higher UA levels (HR: 3.191, 95% CI: 1.456–6.993, *P* = 0.004).

**Table 3:** Univariate Cox regression analysis of OS in patients with primary liver cancer

Characteristics	HR (95% CI)	*P*-value
Sex	0.685 (0.362–1.297)	0.246
Age (y)	0.590 (0.237–1.473)	0.259
BMI (kg/m)	1.102 (0.670–1.814)	0.701
Number of immunotherapy medications	0.605 (0.316–1.159)	0.13
BCLC staging	0.546 (0.212–1.407)	0.21
Child–Pugh classification	1.633 (0.8 86–3.008)	0.116
CNLC stage	1.441 (0.560–3.708)	0.449
PLC type	0.820 (0.289–2.329)	0.71
Hepatitis B infection	0.451 (0.184–1.105)	0.082
History of drinking	0.716 (0.240–2.140)	0.55
History of smoking	1.739 (0.604–5.001)	0.305
Immunotherapy drugs	1.150 (0.593–2.228)	0.679
Targeted therapy	0.778 (0.255–2.371)	0.659
AFP	0.986 (0.497–1.953)	0.967
Number of liver lesions	1.429 (0.770–2.650)	0.258
Vascular invasion	2.750 (0.524–14.421)	0.231
Lymphadenectasis	1.350 (0.740–2.461)	0.328
Distance transfer	1.164 (0.637–2.129)	0.622
Portal hypertension	1.582 (0.434–5.765)	0.487
Venous cancer emboli	0.489 (0.099–2.419)	0.381
Hepatocirrhosis	0.624 (0.214–1.819)	0.387
Ascites	0.999 (0.410–2.434)	0.998
Splenomegaly	2.299 (0.660–8.004)	0.191
Uric acid	3.191 (1.456–6.993)	0.004

To exclude the influence of the confounding factors, after adjusting the test level to P < 0.15 according to clinical practice, variables with *P* < 0.15 in univariate analysis were included in the Cox multivariate proportional regression model (Table 4). Serum UA levels were found to represent an independent prognostic factor for immunotherapy in patients with PLC (HR: 3.131, 95% CI: 1.766–5.553, *P* < 0.001) but were insufficient to predict patient tumor stage, vascular invasion, portal hypertension, and distant metastasis. In addition, the number of immunotherapy doses (HR: 0.543, 95% CI: 0.325–0.907, *P* = 0.02) and Child–Pugh classification (HR: 1.617, 95% CI: 1.110–2.354, *P* = 0.012) were independent prognostic factors for OS in immunotherapy patients with PLC.

**Table 4:** Multivariate Cox regression analysis of OS in patients with primary liver cancer

Characteristics	HR (95% CI)	*P*-value
Number of immunotherapy medications	0.543 (0.325–0.907)	0.020
Child–Pugh classification	1.617 (1.110–2.354)	0.012
Hepatitis B infection	0.778 (0.451–1.340)	0.365
Uric acid	3.131 (1.766–5.553)	0.000

## Discussion

Tumor immunotherapy, such as ICIs and tumor vaccines, has become the main development direction of malignant tumor treatment. With the increasing number of new immunotherapy and combination therapy methods, how to select the most appropriate treatment scheme for patients with malignant tumors remains an urgent problem to be solved. Biomarkers such as PD-L1 expression levels, TMB, MSI, or tumor inflammation can not only help us screen populations who can benefit from immunotherapy but also avoid unnecessary costs and possible severe toxicity in those unresponsive to treatment. Researchers have begun to explore biomarkers to achieve precision immunotherapy. UA, as the end product of purine metabolism, is central to human diseases. UA may originate from the body or from the catabolism of food purines. Recently, studies have reported a possible correlation between UA and the efficacy of anti-tumor therapy in patients with malignant tumors, indicating that high UA levels in the blood may predict worse efficacy of anti-tumor therapy in patients [5]. However, the relationship between UA and the efficacy of anti-tumor therapy in patients with PLC, particularly the correlation with the efficacy of immunotherapy, remains elusive.

In this study, we retrospectively analyzed 99 patients with PLC admitted to our hospital from January 2019 to June 2022. Among these patients, 46 did not return to our hospital after only one dose of immunotherapy, and 53 received two or more doses of immunotherapy. Therefore, the dynamic changes in serum UA level after immunotherapy in patients with PLC could not be monitored, and only the predictive value of serum UA level before immunotherapy on the efficacy of immunotherapy in patients with liver cancer was analyzed. Additionally, targeted therapy has become the first-line therapy for advanced liver cancer. In our cases, 74 patients who received combined targeted therapy and only 25 patients who received immune drug monotherapy were analyzed, which showed no statistical difference between the low and high UA groups, and the combined targeted therapy had no significant effect on the survival of patients with liver cancer. Therefore, patients who received targeted drugs were not excluded from this study.

Many epidemiological studies have linked changes in serum UA with the incidence of malignancy; however, few studies have investigated the relationship between serum UA levels in healthy populations and in patients with PLC. The analysis of this study found that the serum UA level of patients with PLC was higher than that of healthy people. After subgroup analysis, only the UA level of men with liver cancer was higher than that of healthy men, whereas no such correlation was found in women. Similar results have been found in many retrospective and prospective studies of malignancies. Indeed, Dai et al. conducted a prospective study and found that the risk of UA kidney cancer increased by 45% in a population with higher serum UA levels compared with a population with lower UA levels, indicating a correlation between serum UA concentration and cancer risk; after gender subgroup analysis, only the UA levels of women were associated with cancer risk, while the UA levels of men were not [6]. Similarly, a 5-year prospective study by Mi et al. also reported that elevated UA levels were positively associated with the risk of rectal cancer in women, while no association was found in the male population. Additionally, studies have found that increased UA levels may cause an inflammatory stress response and stimulate various transcription factors to promote cell proliferation and migration, thus leading to the transformation of normal quiescent cells into highly invasive cancer cells [5]. After up to 25 years of observational follow-up in a population of 493,281 subjects, Yiu et al. observed that serum UA levels were associated with a higher risk of malignancy [7]. Similarly, a meta-analysis of 12 prospective studies enrolling a total of 632,472 subjects found that high serum UA levels were associated with an increased risk of total malignancy [8]. More studies have found that genetically determined lifelong high serum UA exposure is more harmful than transient high serum UA; high serum UA is often seen in later life, and lifelong high serum UA leads to a higher risk of malignancy and all-cause mortality [9].

Generally, the incidence of PLC is higher in men than in women, with a male-to-female ratio of approximately 2–3:1. In this study, 86 patients were male and only 13 were female. The reason for this may be due to the small number of female patients, which means that the effect of UA levels on the risk of liver cancer cannot be analyzed in this population. Alternatively, perhaps the effect of serum UA levels on the risk of liver cancer in women is smaller than that in men, but the specific mechanism is unknown, which may be due to the difference in sex hormones, body fat content, and metabolic activities between different sexes. Additionally, most male patients have HCC, and most women have ICC, so the differences may also be due to the effect of serum UA level on the incidence of HCC being greater than that on ICC, but more studies are needed to confirm this view. Although we use the large-scale population statistics of the medical reference range as a healthy population serum UA level, these references vary between inspection equipment and the literature and cannot fully fit the regional characteristics of a healthy population; therefore, there remains a need to collect additional information on the regional UA levels of the healthy population.

It also remains to be determined why the serum UA level of patients with liver cancer is higher than that of the healthy population. One possible explanation is that tumor progression is related to cell renewal and apoptosis, where rapid cell renewal [10] and increased purine metabolism by xanthine oxidase (XOD) [11] lead to increased serum UA levels. Another possible explanation is the increased serum UA level in the body due to the increased oxidative response in the presence of the tumor [12]. Indeed, research has found that overcoming hypoxic conditions is crucial for the progression and survival of solid tumors [13]. However, the mechanism responsible for the effect of UA on the risk of PLC remains unclear. During the interaction between UA and the immune system, the inflammation caused by UA crystals and the production of reactive oxygen species (ROS) are considered potential mechanisms to stimulate the growth of cancer cells. It is been demonstrated that ROS is associated with cell damage and malignancy [14]. Mitochondria, DNA, RNA, lipids, and proteins in cells can be disrupted by high levels of ROS, and continuous exposure to inflammatory stimuli may also lead to the formation of local immunosuppression in the liver, providing a relatively tolerant liver microenvironment allowing the survival and growth of tumor cells [15]. Therefore, high levels of serum UA may be a factor in predicting the presence of a tumor due to the relationship between serum UA and the body’s oxidative reaction.

In this study, we collected data on 99 patients with PLC receiving immunotherapy and detected UA results from the first visit to our hospital, with all patients having received immunotherapy within a week. Therefore, in this cross-sectional study, we analyzed the serum UA level and clinical characteristics of 99 patients at diagnosis and found that the serum UA level at diagnosis was significantly associated with BMI and Child–Pugh classification. It has been shown that UA levels increase with stage in head and neck malignancies and PLC, and this phenomenon is particularly significant in patients with advanced breast cancer [16, 17]. Some studies have observed that serum UA levels are positively correlated with C-reactive protein (CRP) and carcinoembryonic antigen (CEA) in patients with metastasis, and increased serum UA may predict metastasis in patients with rectal cancer [18]. However, this phenomenon was not found in this study. In addition, we observed that serum UA levels increased with age, which is consistent with previous reports in the literature that serum UA concentration is affected by aging [19]. Because high serum UA is a symptom, lifestyle factors may contribute to high UA. Obesity can promote an increase in serum UA levels, and the results of this study showed that high serum UA levels and BMI verified this view [20].

The increase in UA level is the result of disordered purine metabolism. Previous studies have reported an association between UA and malignancy; however, the findings are inconsistent. For example, Ames et al. hypothesized that UA, as a powerful antioxidant and a scavenger of free radicals, can inhibit lipid peroxidation at high concentrations and exert antitumor effects [21]. It has also been demonstrated that UA can promote the development of inflammation and plays a key role in the development of malignant tumors [22]. A growing number of recent studies have found that high serum UA levels are associated with increased mortality in patients with malignant tumors [9, 23], especially those of the digestive system. A Chinese study showed that elevated serum UA levels led to increased mortality in hypertensive people with malignancies of the digestive system, while similar findings were reported in patients with non-nonmalignant tumors [24]. Additionally, there have been reports associated with high serum UA levels and poor prognosis in patients with acute myelogenous leukemia [25]. Patients with diffuse large B-cell lymphoma with high serum UA levels showed poor survival outcomes compared with those with lower UA levels [26]. Regarding the respiratory system, patients with NSCLC with higher serum UA levels have higher rates of brain metastases and lower OS [27]. Accumulating evidence supports the potential role of UA metabolism in the pathogenesis of malignancy, including UA-induced inflammation and the production of ROS. Elevated serum UA levels in hyperuricemia mice reduce the effectiveness of immunotherapy in delaying the growth of malignant melanoma [28]. It has been shown that inflammation and oxidative stress can promote tumor cell proliferation and angiogenesis, further causing invasion and metastasis [29]. Therefore, the serum UA level may be a good predictor of immunotherapy outcomes in patients with PLC. In this study, the ROC curve was used to determine the optimal cut-off value of serum UA before immunotherapy to be 259 µmol/l. Based on this value, the patients were divided into high and low UA groups for survival analysis. The results showed that high serum UA levels before receiving immunotherapy were associated with poorer OS. The median survival time in the high serum UA group was 151 days, compared to 312 days in the low serum UA group, and the accumulation of serum UA in vivo may lead to tumor progression and shorten patient survival. Univariate and multivariate analyses showed that serum UA level was an independent prognostic factor for the efficacy of immunotherapy in patients with PLC but was insufficient to predict tumor stage, vascular invasion, portal hypertension, and distant metastasis in patients. Univariate analysis showed that other indicators showed no significant association with patient outcomes. Later, the test level was adjusted to *P* < 0.15 according to clinical practice, and serum UA level, number of immunotherapy drugs, infection of hepatitis B, liver function, and Child–Pugh classification were included for multifactor analysis to avoid the omission of important risk factors. The results showed that in addition to serum UA, the number of immunotherapy drugs used and Child–Pugh classification were also independent prognostic factors for OS in patients with PLC undergoing immunotherapy.

Shi et al. found that apoptotic cells and their antigens together release UA, which can stimulate dendritic cell maturation and activate the immune system, especially CD8 + T lymphocytes [11, 12]. Lymphocytes play a key role in tumor defense by inducing cytotoxic cell death and inhibiting tumor cell proliferation and migration [30]. According to these findings, elevated UA levels should be associated with a better prognosis. The findings of Dziaman et al. are also consistent with the above results, where an increase in UA levels was accompanied by a prolonged survival time in patients with colorectal cancer [31]. However, this is contrary to the findings of this study, in which elevated UA levels before immunotherapy were associated with shorter OS in patients with PLC. Meanwhile, the results of Finish et al. showed a positive correlation between hyperuricemia and increased incidence and mortality of malignancy [5]. Shin et al. included 118 patients with advanced malignancy and found that high serum UA levels were significantly associated with shorter survival times of patients and that UA level was an independent prognostic factor [32]. In patients with renal cell carcinoma, some studies have found that the OS and recurrence-free survival of patients with high levels of postoperative serum UA were shorter than those of patients with low UA, and recurrence-free survival was significantly improved within 5–10 years after patients with reduced postoperative serum UA levels [33]. These are in line with our findings. Moreover, 46% of the patients included in this study only received one course of immunotherapy and did not continue treatment, so the dynamic changes in serum UA level after immunotherapy in patients with liver cancer were not monitored, and only the predictive value of serum UA level before immunotherapy for immunotherapy efficacy in patients with PLC was analyzed. In the future, we should collect more data to study the fluctuation in serum UA levels in patients with PLC before and after immunotherapy and how to use serum UA level to screen people who benefit from immunotherapy. By reviewing the previous literature, we found that the relationship between UA and malignant tumor treatment efficacy is not exact; therefore, we should conduct in-depth research on the pathophysiological mechanism, intending to be able to measure serum UA levels to predict tumor treatment efficacy by malignancy.

With the application of dozens of anti-tumor or adjuvant antitumor drugs in the clinic, an increasing number of patients develop therapeutic resistance. Given that the development of new drugs requires long-term, expensive research, drug repurposing is becoming a new strategy for treating treatment. Existing drugs, such as statins and aspirin, are used to treat cardiovascular diseases and have now received widespread attention for treating malignancies [34, 35]. Many studies have reported the potential role of UA in the development of malignant tumors, so perhaps UA-lowering drugs also have some anti-tumor potential. However, the UA-lowering measure was not used in the patients enrolled in this study, so the possible anti-tumor effect exerted by the UA-lowering drugs could not be analyzed. The potential antitumor effects of UA-lowering drugs are discussed only along with previous studies. Drugs that disrupt microtubule dynamics are widely used in chemotherapy for malignancy. Given their importance in mitosis and spindle formation, microtubules have long been recognized as one of the ideal targets for antitumor therapy [36]. Indeed, drugs with microtubule-damaging activity, such as colchicine, are often used to improve acute gout attacks, and studies have begun analyzing their potential antitumor activity [37]. Colchicine, by binding to co-binding sites on β -tubulin, suppresses microtubule polymerization, resulting in cell mitosis remaining in metaphase, thus exerting antitumor effects as an inhibitor of microtubule activity [38]. Additionally, rearranged during transfection (RET) during protein expression was found to lead to tumor enlargement, resulting in a later patient stage, while colchicine selectively binds to RETg, perhaps exerting an antitumor effect [39]. It has been reported that colchicine can be used as an anti-tumor agent in the form of nanoparticles to inhibit the growth of colon and liver cancer cells [40]. The liver is a common site of metastasis in many malignancies, and nonalcoholic fatty liver disease (NAFLD) may be an important factor in tumor metastasis to the liver [41, 42]. UA is central to the development of NAFLD through the nucleotide-binding NOD-like receptor protein 3 (NLRP 3) inflammasome regulating hepatic steatosis and insulin resistance [43]. It has also been shown that allopurinol reduces the activation of NLRP 3 by lowering UA. Considering the role of allopurinol in alleviating NAFLD, allopurinol can also prevent malignant tumor metastasis to the liver [44]. It has been demonstrated that allopurinol can reduce blood glucose and induce ROS to reduce hepatic oxidative stress, inflammation, and steatosis, thereby reducing NAFLD risk. In contrast to allopurinol, in the NAFLD mouse model, febuxostat reduced UA levels and xanthine oxidase activity and more effectively reduced insulin resistance, lipid peroxidation, and liver inflammation, suggesting that febuxostat plays a more efficient role in preventing liver metastasis [45].

This study has several strengths. Its main advantages are the collection of the PLC population receiving immunotherapy, long follow-up time, affordable UA testing, and widespread clinical use. This study also has several potential limitations that warrant discussion. As with other retrospective studies, the effects of selection bias, incomplete data collection, and the inability to review patients’ dietary habits cannot be excluded. Given the strict inclusion and exclusion criteria, the number of patients was also limited. The included patients with PLC lack long-term follow-up data, making it impossible to monitor the UA fluctuations in each patient to better study the predictive value for the efficacy of immunotherapy. Moreover, this is a single-center study that limits the researchers’ ability to explore the mechanisms of the association between UA and immunotherapy for liver cancer; thus, further longitudinal studies are needed to compensate for this. Some confounding factors associated with UA, such as diet, exercise, and alcohol consumption, were not included as variables in the multiple regression analysis, where more clinical parameters were needed to explain the relationship between serum UA and metastasis status. Education, environmental differences, and economic income level may also be confounding factors; however, these factors could not be assessed in this study. The role of elevated serum UA levels as an independent risk factor for the occurrence and development of malignancy remains controversial, which may vary by sex. Elevated UA levels may be a valuable long-term surrogate marker rather than an independent risk factor or even a carcinogen itself, as increased UA levels indicate lifestyle factors. Therefore, large-scale prospective and multicenter studies are needed to validate the results presented here. How to predict the efficacy of PLC immunotherapy requires further study. Despite these limitations, our findings suggest that serum UA may be a novel marker for the primary prediction of the prognosis of immunotherapy in patients with PLC.

## Conclusions

This study showed that the serum UA level is a reliable biomarker for predicting the prognosis of patients undergoing immunotherapy for PLC, and might provide a basis for the individualized treatment of these patients. Dynamic monitoring of the serum UA level may compensate for the deficiency of the current liver cancer staging system.

## Data Availability

The datasets generated for this study are available on request to the corresponding author.

## Abbreviations

AFP: Alpha-fetoprotein
ALT: Alanine transaminase
AST: Aspartate transaminase
AUC: Area under the curve Barcelona Clinic Liver Cancer
BMI: Body mass index
CEA: Carcinoembryonic antigen
CI: Confidence interval
CNLC: China liver cancer classification
CRP: C-reactive protein
ECOG: Eastern Cooperative Oncology Group
HCC: Hepatocellular carcinoma
HR: Hazard ratio
ICC: Intrahepatic cholangiocarcinoma
ICI: Immune checkpoint inhibitor
MSI: Microsatellite instability
NAFLD: Nonalcoholic fatty liver disease
NLRP 3: NOD-like receptor protein 3
OS: Overall survival
PD-L1: Programmed death receptor ligand-1
PLC: Primary liver cancer
RET: Rearranged during transfection
ROC: Receiver operating characteristic
TMB: Tumor mutational burden
UA: Uric acid
ULN: Upper limit of normal
